# Nonlinear thermal lensing of high repetition rate ultrafast laser light in plasmonic nano-colloids

**DOI:** 10.1515/nanoph-2021-0775

**Published:** 2022-01-18

**Authors:** Leonidas Agiotis, Michel Meunier

**Affiliations:** Laser Processing and Plasmonics Laboratory, Department of Engineering Physics, Polytechnique Montréal, C.P. 6079, succ. Centre-ville, Montréal, QC, H3C 3A7, Canada

**Keywords:** femtosecond laser, high repetition rate, plasmonic nanoparticles, self-channeling, self-trapping, thermal lensing

## Abstract

We report on experimental observations of phenomenological self-trapping in plasmonic colloids of varying plasmon peaks in the visible/near infrared. A femtosecond (fs) oscillator is used in both pulsed (35 fs, 76 MHz) and continuous wave (cw) operation for comparison. We show that for both modes and for all examined colloids (and under typically applied external focusing conditions in self-trapping studies in colloidal media) nonlinear propagation is governed by thermal defocusing of the focused beam, which precedes the steady-state regime reached by particle diffusion, even far from the plasmon resonance (or equivalently for non-plasmonic colloids, even for low absorption coefficients). A strategy for the utilization of high repetition fs pulses to mitigate thermal lensing and promote gradient force-induced self-trapping is discussed. Notably, nonlinear thermal lensing is further accompanied by natural convection due to the horizontal configuration of the setup. Under resonant illumination, for both fs and cw cases, we observe mode break-up of the beam profile, most likely due to azimuthal modulation instability. Importantly, time-resolved observations of the break-up indicate that in the fs case, thermal convection heat transfer is reduced in magnitude and significantly decoupled in time from thermal conduction, presumably due to temperature increase confinement near the particles. We anticipate that our findings will trigger interest toward the use of high repetition fs pulses for self-channeling applications in nano-colloids.

## 1 Introduction

Nonlinear self-trapping of laser light in soft-matter systems, such as dielectric [1], [2], [3], [4], [5] or plasmonic colloids [6], [7], [8], [9], [10], [11], [12], [13], [14], [15] as well as biological media [16], [17], [18], [19], [20], has attracted increased attention over the past decade. The effect is described as diffraction-less propagation of laser light, trapped over many diffraction lengths by virtue of the intensity-dependent nonlinear refractive index of the medium. Indeed, the possibility has been noted of tuning the nonlinear response of soft-matter systems via laser-induced local refractive index modulation, leading to the observation of novel self-action effects. Consequently, soft-matter systems provide a unique platform for the fundamental investigation of nonlinear effects and for prototypical applications based on self-focusing and instability beam break-up [17, 21].

In the case of plasmonic nanocolloids, several studies have reported that self-trapping of laser light is possible by virtue of particle concentration gradients arising from the enhanced particle polarizabilities and exerted on them optical forces [7, 8, 10, 14, 15]. Others have demonstrated in the same context that the beam is not self-trapped; in fact, a self-channeling effect (a phenomenological self-trapping) is observed because of nonlinear thermal lensing, giving the impression of a self-trapped beam, particularly when the laser field is tuned near the plasmon resonance [6, 9, 13]. In this case, the medium acts as a laser-induced (due to optical absorption by the particles) thermal lens, which tends to collimate the externally focused beam, much like an optical telescope. Thus, the conditions that demarcate the dominance of either thermal or particle diffusion (due to optical forces) effects, especially far from the plasmon resonance, in the context of self-channeling in plasmonic colloids remain unclear.

Further, most studies of self-trapping of optical beams have been conducted by use of cw laser sources. Interestingly, under certain focusing conditions, fs laser pulses of high repetition rate can be used to generate quasi-continuous wave interactions due to cumulative effects [22], [23], [24], [25], [26]. Additionally, in the case of plasmonic systems, fs pulses lead to higher localization of thermal effects [27]. Therefore, the use of high repetition fs pulses in plasmonic nano-colloids in this context and how it compares to cw interaction is particularly interesting and has not been explored yet.

The objectives of this work are the following:

1) Study the phenomenological self-trapping (self-channeling) of high repetition rate fs laser pulses in plasmonic nanocolloids of varying plasmon peaks with respect to the incident field wavelength, by applying commonly reported focusing conditions. We show that the effect exhibits characteristics of thermal self-defocusing of a focused beam (for both cw and fs operation) even far from the plasmon resonance and is generalized for any absorbing medium of given thermal properties. We discuss conditions under which optical force-induced self-trapping can be achieved as opposed to nonlinear thermal lensing by means of high repetition rate fs pulses.
2) Explore the features of the observed nonlinear thermal lensing induced by high repetition fs pulses as opposed to cw laser light, under plasmon-resonant interaction. We specifically aim to explore if thermal effects are alleviated under fs illumination. To this end, we analyzed distinct features in the dynamics of a beam spatial mode break-up and thermal distortion (blooming) at high input powers, when resonant samples are excited by either cw or fs illumination, and their association to the thermal response of the nanoparticles.

## 2 Results

### 2.1 Nonlinear thermal lensing (fs pulses)

**Figure 1:**
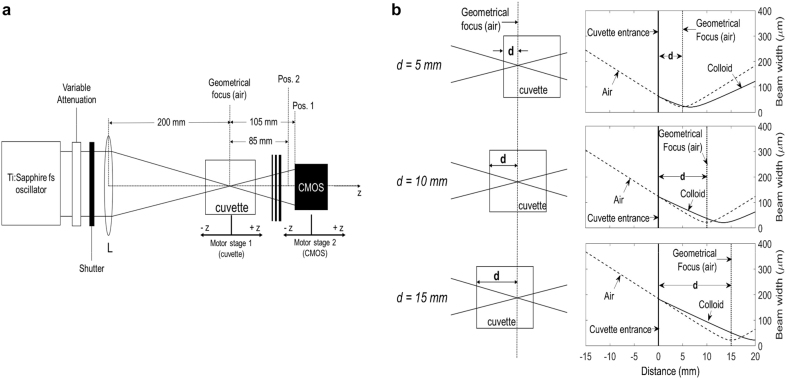
(a) The experimental setup (detailed in Supplementary material). (b) The figures on the left show the three examined cuvette positionings with respect to the geometrical focus of lens L in air, as defined by the parameter *d*. The figures on the right indicate quantitatively the shift of the actual beam waist position inside the 20 mm long cuvette, when filled with the examined colloids (linear regime). This is because of the difference between the refractive index of air (*n*_0_ ≈ 1) and colloids (*n*_0_ ≈ 1.33). The values in air (dashed curves) correspond to experimentally measured beam width along *X*-axis (shown in Figure A1(b), Supplementary material). The values in the colloids (solid curves) have been evaluated by Eq. (A1b) (Supplementary material) for *n*_0_ = 1.33.

A series of experiments were performed to understand the origin of the self-channeling effect under fs illumination in plasmonic nanocolloids. We evaluated the power-dependent full width half maximum (FWHM) far-field beam width and divergence of an externally focused beam as it emerged from a 20 mm optical cuvette that contained each of four examined plasmonic nano-colloids (samples S1, S2, S3, S4 as shown in Table 1). Images of the FWHM far-field beam width were collected by a CMOS camera placed at two different positions in the far-field (Figure 1(a)). A Ti:Sapphire laser in fs operation (wavelength 800 nm, pulsewidth 35 fs, repetition rate 76 MHz) was used. The laser oscillator could run in both fs and cw modes. The initial beam 1/*e*^2^ radius was elliptical, evaluated $w_{0,Y}$ ∼ 2.8 mm along *Y* axis (vertical) and $w_{0,X}$ ∼ 2.4 mm along *X* axis (horizontal). We defined the distance d between the entrance of the cuvette and the beam waist in the medium (Figure 1(b)). Additionally, we performed optical transmittance and *z*-scan measurements on the examined samples under fs illumination. All samples exhibited linear absorption in the range of the applied input powers (1–280 mW) for all examined positions d. The *z*-scan showed negative refractive nonlinearity, presumably due to thermal lensing, governed by the thermo-optical coefficient $\frac{dn}{dT}$ of the solvent (water) for all colloids. All methods are described in detail in the Supplementary material (Sections A1.1 and A1.2). Table 1 summarizes the results of optical characterization.

**Table 1:** Linear absorption and thermo-optic coefficients of the examined plasmonic nanocolloids, characterized by optical transmittance and *z*-scan measurements by fs irradiation at 800 nm ^a^Width × Length. ^b^Diameter. ^c^Longitudinal, ^d^Transverse.

Sample	Average size (nm)	Surface plasmon resonance wavelength (nm)	$a\;\left(\lambda_{0}=800\;\mathrm{nm}\right)\;\left(cm^{-1}\right)$	$\frac{dn}{dT}\;\left(\frac{10^{-5}}{\mathrm{{}^{\circ}C}}\right)$
S1 (Au nanorods)	10×38 ^a^	780^c^, 510^d^	2.10±0.10	−3.2±0.4
S2 (Au nanorods)	10×50 ^a^	900^c^, 510^d^	0.84±0.09	−2.7±0.4
S3 (Au nanospheres)	50 ^b^	525	0.24±0.05	−2.8±0.3
S4 (Au–Ag alloy:15–85 nanospheres)	40 ^b^	450	0.06±0.02	−2.9±0.3

**Figure 2:**
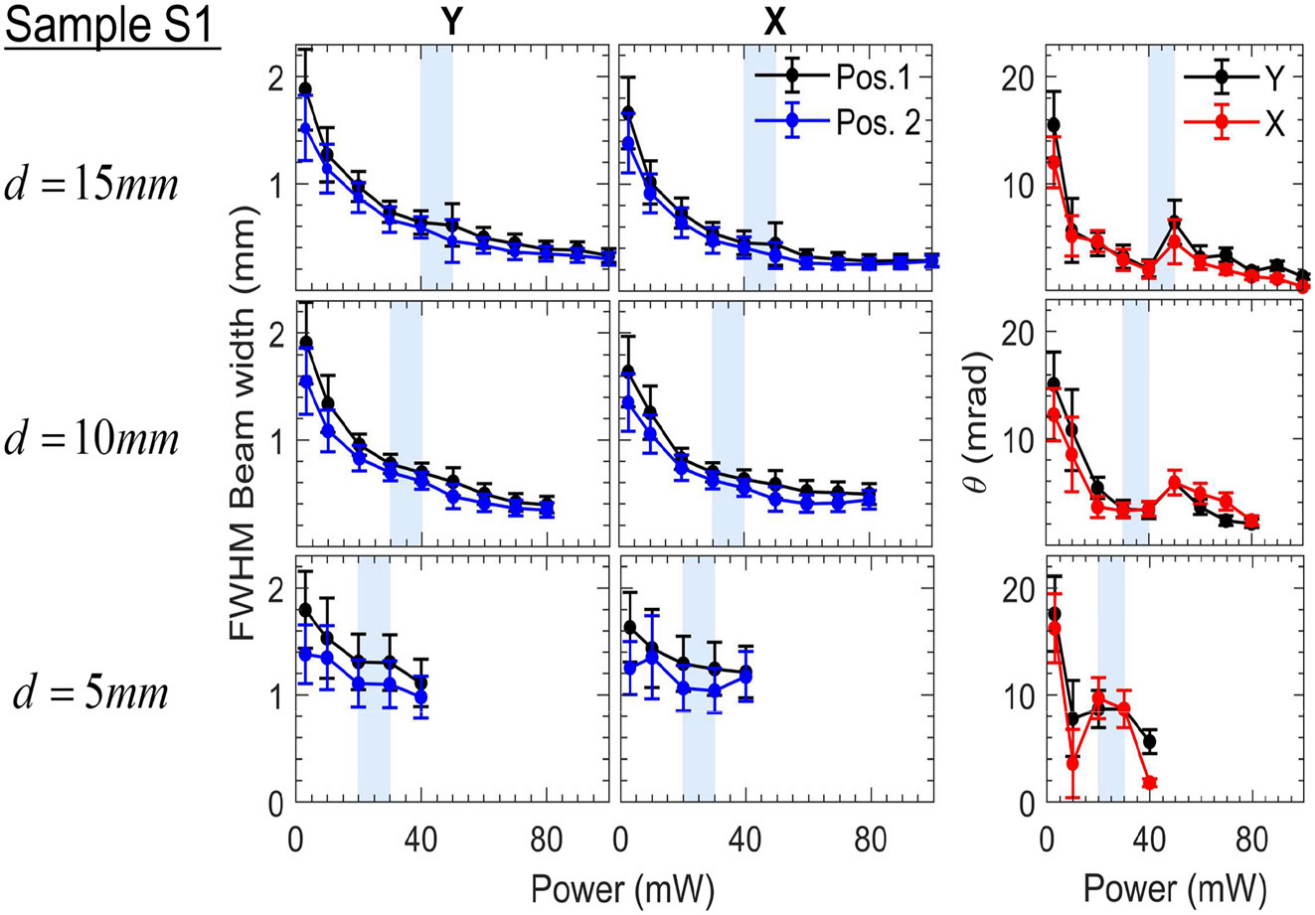
Experimental measurements of the far-field FWHM beam width and divergence θ for sample S1 as a function of power for three different values of d (15, 10 and 5 mm). The beam width was determined at two different positions in the far field (shown in Figure 1(a) and in Figure A1(a) of the Supplementary material) to evaluate θ. Results over both *Y* and *X* axis are presented. The shaded, light-blue areas indicate the observed power onset of Airy function-type diffraction interference on the beam profile.

First, we examined the influence of the position parameter d in the case of the resonant sample S1. Three values of d were examined, summarized in Figure 2. Initially, at low power and for all cuvette positions, the FWHM beam width was ∼1.9 mm at the *Y* direction and ∼1.6 mm at the *X* direction at a distance ∼10.5 cm (position 1) away from the focus, and ∼1.6 mm at the *Y* direction and ∼1.4 mm at the *X* direction at a distance ∼8.5 cm (position 2) away from the focus, which yields a divergence ∼15 mrad.

The following qualitative observations can be made: as the input power increased, the beam width gradually decreased for all cuvette positions, retaining a nearly Gaussian profile. The behavior continued up to a critical power value where a diffraction ring was formed on the background, presumably because of strong thermal aberration (phase-front spatial interference of Airy function-type). The onset of this transition was recorded and is shown in Figure 2 marked by a shaded, light-blue area.

The FWHM of only the central Airy disk was evaluated at higher powers than the onset of the foresaid transition. The central Airy disk was seen to gradually shrink and decay at increased input powers (>40 mW for d=5 mm, >80 mW for d=10 mm and >110 mW for d=15 mm) while outer rings gained higher radiation densities. Thus, estimation of its FWHM was not performed beyond these powers. In addition, convection currents arose as the liquid was heated, resulting in a downward beam deflection, which became more pronounced as the input power increased.

We make the following quantitative evaluations on the beam width and the divergence of the beam as a function of input power (<100 mW) for all three examined d values (shown in Figure 2): For d=5 mm the FWHM beam size obtained values >1 mm at the far-field. When the focus was positioned deeper inside the cuvette, a smaller minimum beam width was obtained (down to ∼350 μm for d=10 mm and ∼250 μm for d=15 mm).

The divergence of the beam for d=15 mm exhibited rapid three-fold decrease, from ∼15 mrad to ∼5 mrad within ∼1–10 mW. At higher powers, it decreased on average down to ∼8 mrad when d=5 mm and to ∼2–4 mrad when the focus was positioned deeper in the cuvette. Beyond the onset power of Airy-type interference, the divergence was seen to monotonically decrease for all positions. Specifically, for d=15 mm, both divergence and spot size attained overall minimum values (∼1 mrad and ∼280 μm, respectively). Conclusively, the minimum values of divergence and beam size were higher as the focus was located closer to the input of the cuvette.

**Figure 3:**
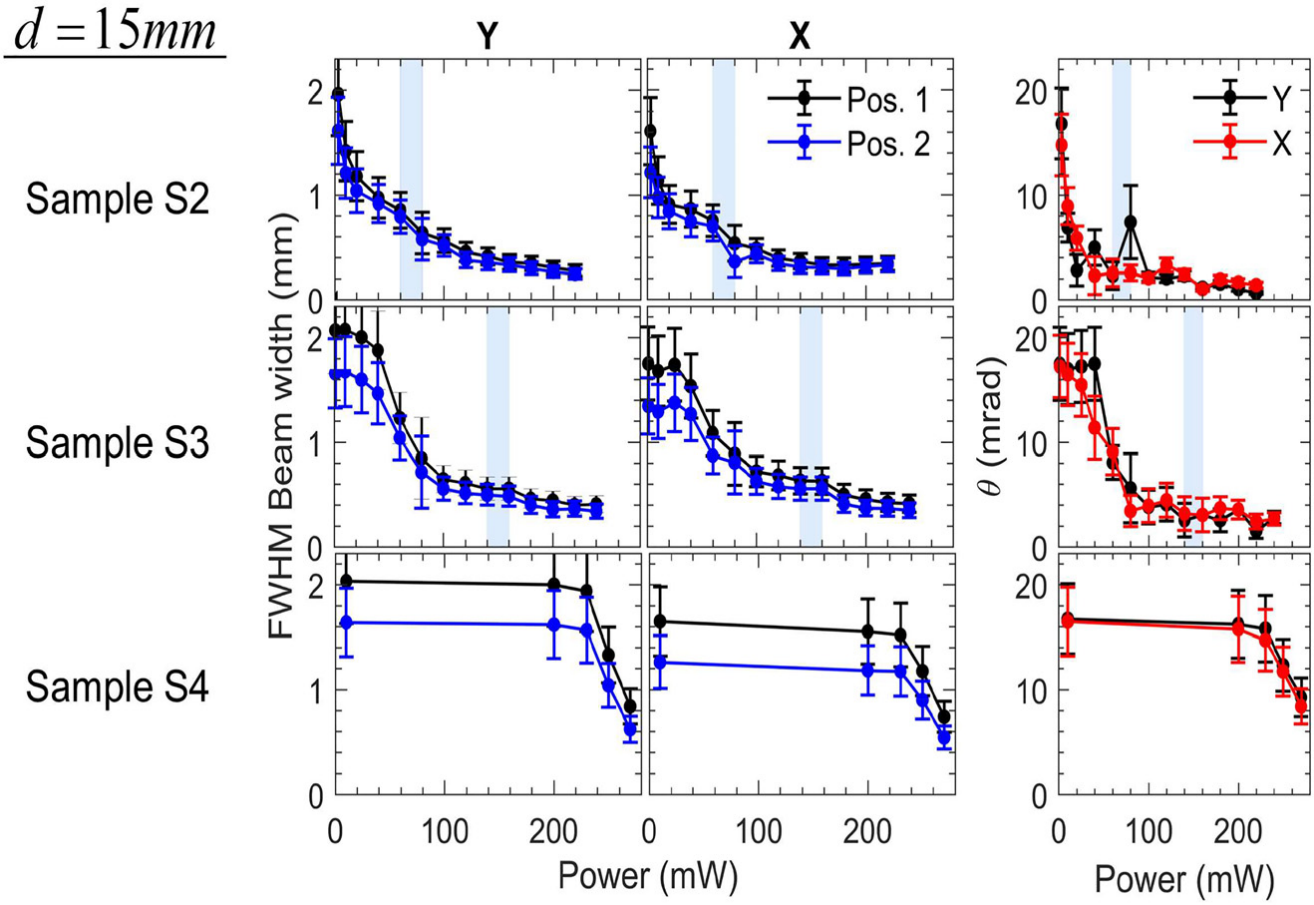
Same as Figure 2, for the samples S2, S3 and S4 and for d=15 mm.

For the rest of the samples, we performed experiments for d=15 mm. The choice was based on the observed minimization of the divergence and beam width for sample S1. The results are shown in Figure 3. Similar features of the nonlinear thermal lens were observed for each sample. For comparison, the onset for observation of thermal aberration Airy-type interference (diffraction rings) for sample S2, required ∼1.5× higher power compared to S1, while for sample S3 a ∼3.5× power increase relative to S1 was needed. Notably, the minimum values of far-field beam width and divergence are evaluated to be smaller as the excitation wavelength is closer to the resonance of the samples (i.e., for higher absorption coefficient).

**Figure 4:**
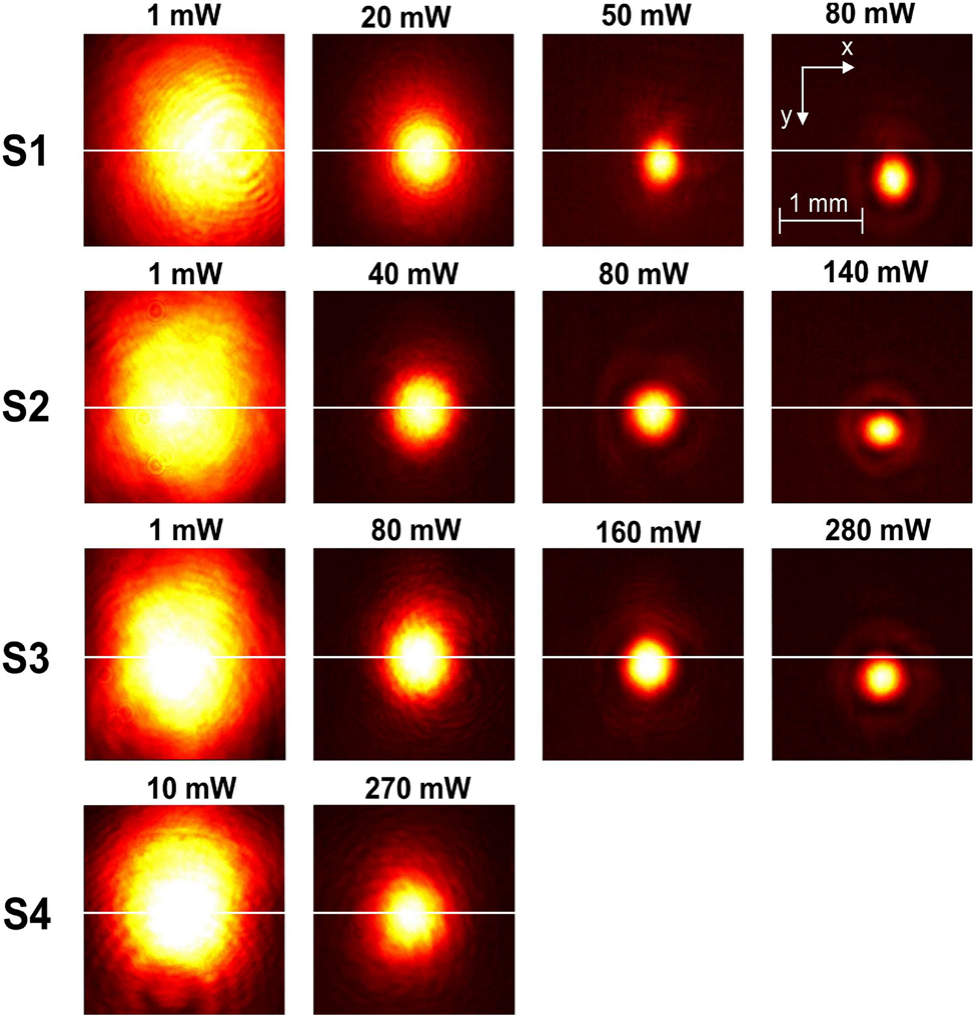
Far-field beam width profiles under fs excitation of samples S1, S2, S3 and S4 for various optical input powers recorded at position 1 and d=15 mm. The figure demonstrates similar behavior for all samples: the first column shows the initial profile, the second column shows the required power for shrinking of the beamwidth to approximately half of the initial, the third column shows the appearance of Airy-type diffraction interference, and the fourth column shows further shrinkage of the central Airy disk and downward displacement δy of the beam profile due to convection currents. The horizontal line shows the initial position of the beam center on the *Y* direction. The inset scale and axes apply for all figures. The *x* axis is horizontal, and the *y* axis is vertical and pointing downwards to define the positive direction of δy.

For sample S4, it was not possible to determine the onset of Airy function-type interference since not enough power was available by our laser source (<280 mW). However, we observed the characteristic reduction (as described for all other samples) of the far-field beam profile and divergence above ∼220 mW. The power dependencies of the far-field beam width for each sample are qualitatively depicted in Figure 4.

### 2.2 Comparison of resonant nonlinear thermal lensing between fs and cw operation

#### 2.2.1 Nonlinear defocusing

**Figure 5:**
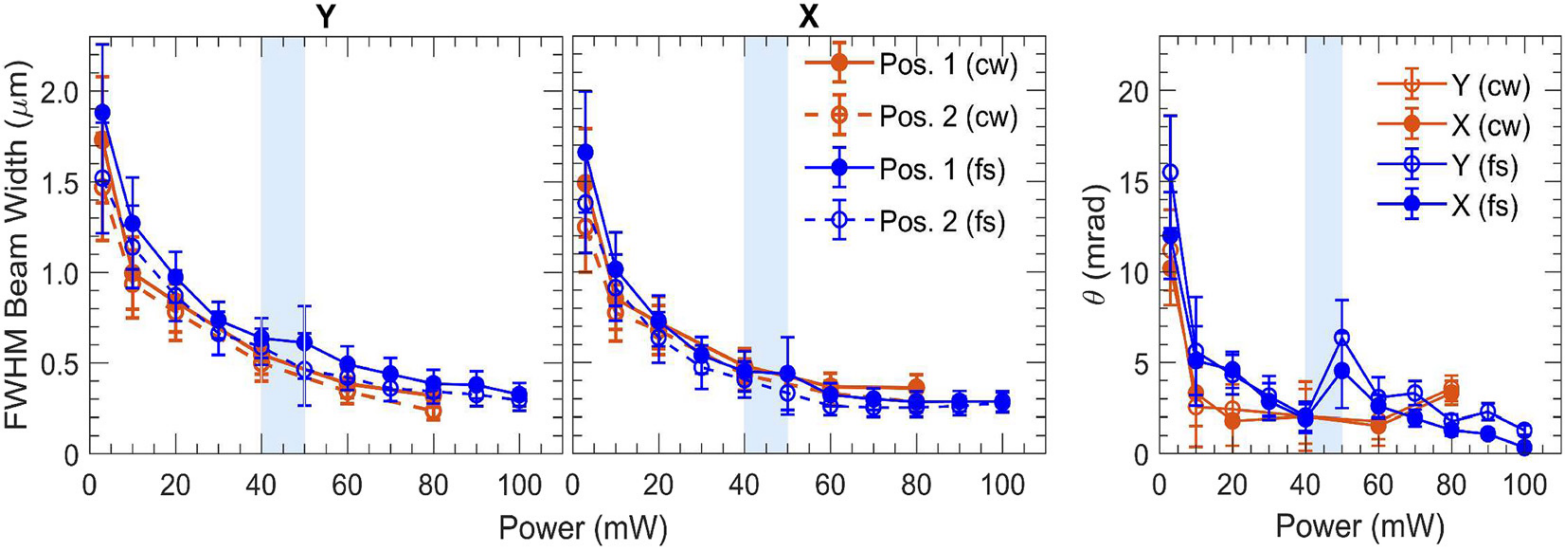
Comparison between use of cw and fs excitation on the experimental measurements of the far-field FWHM beam width and divergence θ for sample S1, as a function of power for d=15 mm.

In cw operation, the resonant sample S1 exhibited increased absorption (15% higher than fs excitation). This is presumably due to the monochromatic excitation of the plasmon mode, as opposed to the spreading of energy over the optical frequencies of the fs spectrum. Indeed, the latter is expected to result in less efficient mode-matching with the surface plasmons. We have performed comparison of the two cases (fs and cw) when d=15 mm (Figure 5).

In cw mode, the FWHM beam width obtained lower values at the same input power compared to the fs case up to ∼30 mW. For higher powers, this trend continued only on the *Y* axis, while in the *X* axis, no significant difference was observed between cw and fs operation beam widths. On the other hand, in the power interval between 3 and 40 mW, the divergence of the beam obtained smaller values in cw operation down to about 1.5–3.0 mrad. Formation of Airy function-type interference was observed above ∼40 mW. At optical power ∼70 mW, the beam divergence increased for cw operation, which opposes the observations of fs operation.

#### 2.2.2 Convection and thermal blooming

**Figure 6:**
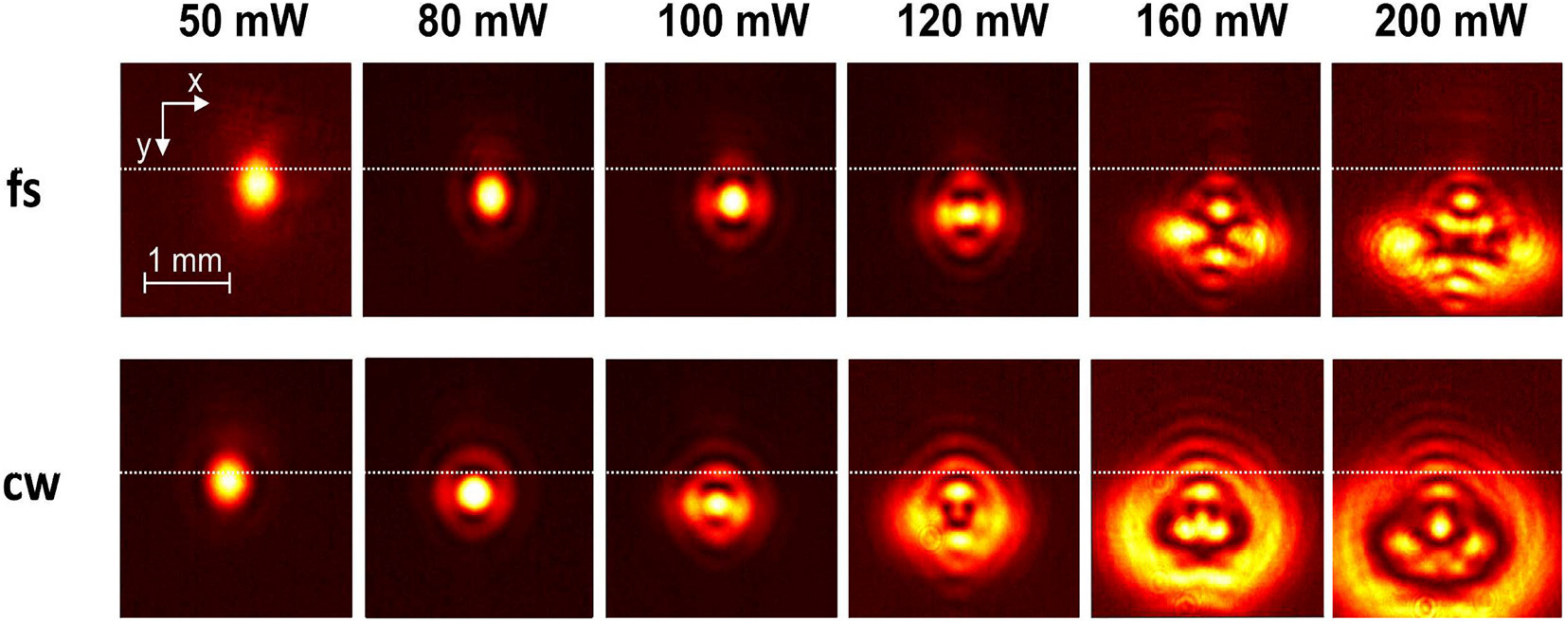
Far-field beam width profile under fs and cw excitation of sample S1 for high input powers recorded at position 1 and d=15 mm. Beam profile break-up effects are observed beyond 120 mW for both cases. Pronounced beam break-up is observed under cw excitation at lower input power and strong thermal blooming beyond 160 mW with a characteristic highly asymmetric lower half-portion. In the case of fs operation, a less asymmetric, yet complex profile is observed. The downward displacement can be compared in the two cases with respect to the low-input-power position of the center of the beam (white horizontal line).

Figure 6 shows images of the profiles at various powers for fs and cw excitation. Evidently, induced convection currents caused a downward deflection of the beam along the *y* axis.

Similar features on a beam profile break-up under cw operation preceded in optical power the ones acquired under fs operation. Specifically, break-up of the first outer ring was observed, at an onset of ∼100 mW and ∼120 mW for cw and fs operation, respectively. The first outer ring clearly breaks up into four bright spots, at ∼120 mW input power for both cases. As the power increased in cw operation, the thermal blooming effect [28] manifested itself (at ∼160 mW). Contrarily, in fs operation the profile retained its axial symmetry along *x* and *y* axes obtaining yet a complex structure, while it was elongated along the *x* axis up to ∼200 mW.

**Figure 7:**
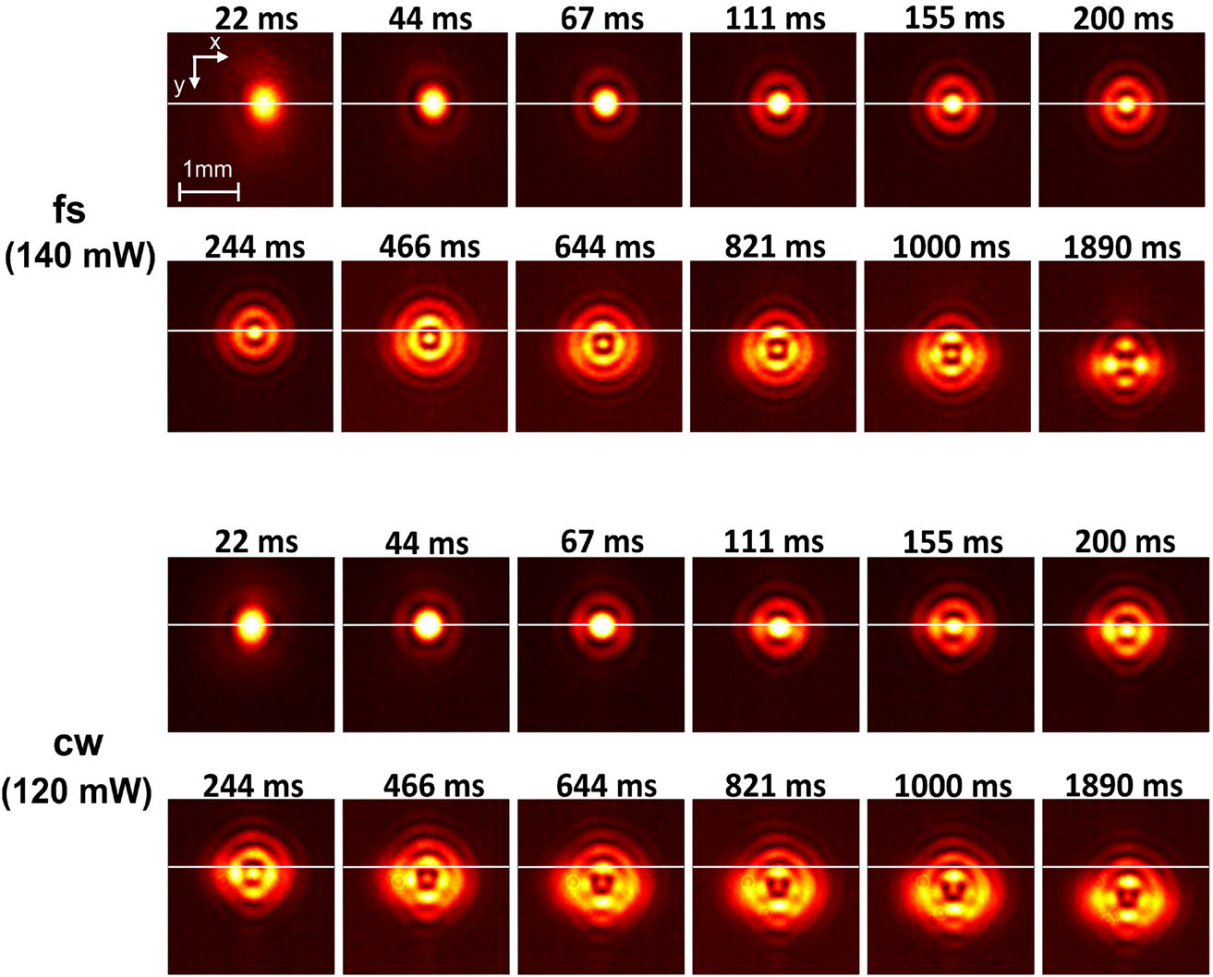
Time-resolved far-field beam width profile under fs and cw excitation of sample S1 for input powers of ∼140 mW and ∼120 mW, respectively, recorded at position 1 and d=15 mm. The selection of the foresaid input power leads to a fair comparison between the two profiles due to the 15% higher absorption coefficient calculated in the case of cw operation. Note the subtle downward displacement δy of the beam core and onset of beam break-up just after 200 ms for the case of cw operation. Contrarily, for fs operation, downward displacement is observed only after ∼466 ms and the onset of beam break-up is observed after ∼821 ms. Finally, a drastic beam profile break-up is observed for the fs case between the time interval of 1000 and 1890 ms (Suppplemental video).

We have obtained time-resolved images of the far-field beam profile (see Methods, Section A1.1 – Supplementary material) for the specific cases of input power of 120 mW and 140 mW in cw and fs pulsed operations, respectively. The images were used to analyze the difference in the dynamics of the mode break-up. Results of images taken for both cases are shown in Figure 7.

For the fs case, observable growth of the break-up of the first outer ring surrounding the decaying core occurred only after ∼1 s as opposed to the cw case for which the same effect was observed after ∼200 ms from the opening of the shutter. Further, the beam profile break-up in the fs case became pronounced after the beam acquired its final position, under convection-induced displacement. In the cw case, the onset of profile displacement subtly preceded in time the one in the fs case (compare for example the central core displacement in the two cases after ∼200 ms and ∼244 ms from the opening of the shutter). Finally, the break-up was observed to be typically accompanied with stochastic, small-scale motion of the profile around the center of the beam (Supplementary video). For the case of cw excitation, such stochastic motion was observed as early as ∼200 ms, and was generally more pronounced, whereas, in the fs case it became observable only after ∼1 s from the opening of the shutter.

## 3 Discussion

### 3.1 Nonlinear thermal lensing (fs pulses)

**Figure 8:**
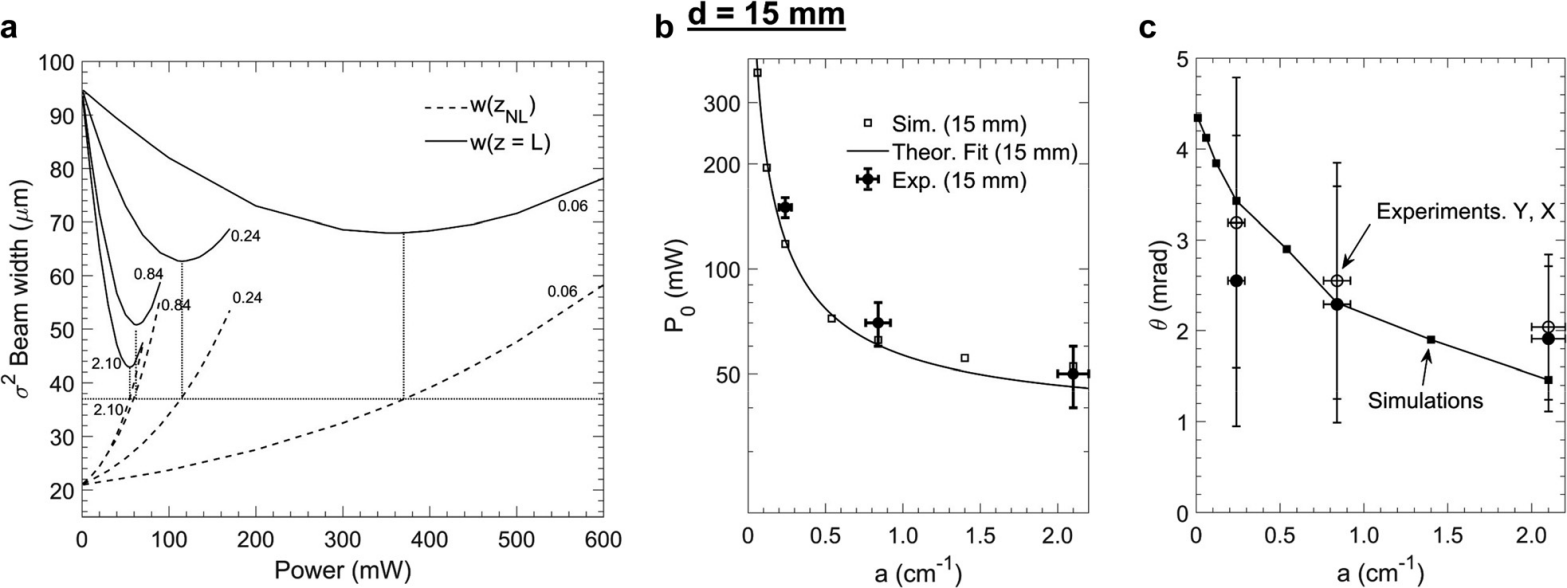
Results of numerical simulations based on the nonlinear Schrödinger equation with a thermal nonlinearity (Eqs. (A3) and (A4), Supplementary material) and comparison with experimental observations for d=15 mm. (a) Numerical evaluation of the $\sigma^{2}$ beam width at the output w(z=L) (solid curves) and of the $\sigma^{2}$ nonlinear beam waist $w\left(z_{NL}\right)$ (dashed curves) as a function of P. The calculations correspond to the values of absorption coefficient a of the examined samples (in $cm^{-1}$, shown close to each curve). Dotted lines indicate that at the inflexion points of w(z=L) (i.e., at $P=P_{0}$) the nonlinear beam waist $w\left(z_{NL}\right)$ is stretched by the same factor m∼1.75 compared to the linear beam waist $w_{f}=21\;\mu m$. (b) Comparison of $P_{0}$ (a) by numerical simulation (squares), fitting of analytical expression (2) for *m* = 1.75 (solid line) and experimental values (circles). (c) Comparison of θ(α) between numerical simulations (squares) and experimental measurements (circles).

The phenomenological self-trapping observed experimentally was compared theoretically to a model based on the stationary nonlinear Schrödinger equation with thermal nonlinearity [Eqs. (A3) and (A4), Supplementary material]. We first evaluated $\sigma^{2}$ near-field beam width for d=15 mm as a function of P after L=30 mm of propagation in media characterized by absorption coefficient a, thermal conductivity κ and thermo-optic coefficient dn/dT (Supplementary material, Section A.1.3). The results are shown in Figure 8(a) for the four different values of a that correspond to the nano-colloids examined experimentally and for κ, dn/dT of water. The $\sigma^{2}$ near-field beam width at the output w(z=L), exhibits a parabolic behaviour as a function of P, quantitatively different for each a. An inflexion point is formed at an optimum power $P_{o}\left(a\right)$ that demarcates thermal aberration in the far-field. The inflexion point has a simple physical interpretation. The initial phase-front curvature of the focused beam, which reads $\delta \varphi_{i}=\frac{\pi }{\lambda R}w_{0}^{2}$ (R, $w_{0}$, denote the radius of curvature and beam width at the input, respectively), is compensated by the thermal self-induced phase. The latter is estimated as $\delta \varphi_{T}\left(r\right)=\frac{\pi }{\lambda }\overset{z_{NL}}{\underset{0}{\int }}\frac{\delta n\left(r,z\right)}{n_{0}}dz$, where $z_{NL}\left(P\right)$ is the distance between the entrance of the cuvette and the nonlinear beam waist, and $\delta n\left(r,z\right)=\frac{dn}{dT}\delta T\left(r,z\right)$ (where δT(r,z) denotes local temperature increase), so that the inflexion point appears when $\delta \varphi_{i}\approx \delta \varphi_{T,\max}$. Evidently, in the linear regime, the beam waist is located at $f=n_{0}\times d$ ($n_{0}$ is the refractive index of water), and for $P\to P_{0}$, in the nonlinear regime, the beam waist is moving (increasing) monotonically at $z_{NL}\left(P\right)>f$. We observed that, at the inflexion point ($P=P_{0}$), the $\sigma^{2}$ nonlinear beam waist $w\left(z_{NL}\right)$ was expanded compared to the $\sigma^{2}$ linear beam waist $w_{f}$ in each medium by the same factor m, independently of a, which was estimated $m\equiv w\left(z_{NL}\right)/w_{f}∼1.75$. For $P>P_{0}\left(a\right)$, it holds $\delta \varphi_{T,\max}>\delta \varphi_{i}.$ The beam begins to defocus and as a result the $\sigma^{2}$ beam width at the output w(z=L) increases (Figure 8(a)). The position $z_{NL}$ moves further towards the output of the cuvette for increasing P so the curve of the $w\left(z_{NL}\right)$ gradually approaches w(z=L) (Figure 8(a)). Figure 8(b) summarises the behavior of $P_{0}\left(a\right)$. We repeated the process for d=10 mm with similar observations and determined the corresponding $P_{0}\left(a\right)$ (Supplementary material, Section A2). We further found that in this case m∼1.5, which shows that m depends on $\delta \varphi_{i}$, i.e., the initial focusing condition, but not on a.

Importantly, $P_{0},$ for which it holds $\delta \varphi_{i}\approx \delta \varphi_{T,\max}$, can be estimated experimentally by recording the value of P *that coincides with the appearance of Airy function type diffraction rings in the far-field*. A fair agreement is observed between simulations and experimental values, where $P_{0}$ was further determined numerically for three more a values (1.40,0.54 and $0.12\;cm^{-1}$) in Figure 8(b).

The behavior of $P_{0}\left(a\right)$ can be interpreted in accordance to the theoretical analysis of reference [29] addressing the problem of thermal self-focusing within the aberration-free approximation. The analysis showed that for input power $P_{\mathrm{in}}$, beam compression n is obtained due to positive thermal self-focusing that depends on a critical power $P_{\mathrm{cr},0}$, the diffraction length $L_{d}\equiv z_{R}$ and a of the medium. The foresaid relation reads

(1) $$P_{\mathrm{in}}\approx P_{\mathrm{cr},0}\left(\frac{n^{2}-1}{aL_{d}\;\ln\left(n^{2}\right)}+\sqrt{n^{2}-1}\right)$$

While the problem was solved implying positive nonlinearity, the reduced propagation equation (e.g., Eq. (21) in [29]) is identical for negative nonlinearity if also an external focusing initial condition is considered (${\partial_{z}w|}_{z=0}=-w_{0}/R$), so that n expresses beam waist stretching instead of compression for a given $P_{\mathrm{in}}$. Accordingly, at $P_{0}$, the beam waist stretches by the factor m defined above. By numerical simulations, we used the ansanz of Eq. (1) for the examined problem, and found that $P_{0}$ can be determined, accounting for a fitting dimensionless parameter A, by

(2) $$P_{0}\approx \frac{\kappa k_{0}\lambda^{2}}{n_{0}\left|\frac{dn}{dT}\right|}\left(\frac{A\left(m^{2}-1\right)}{aL_{d}\;\ln\left(m^{2}\right)}+\sqrt{A\left(m^{2}-1\right)}\right)$$

where, we have assumed $P_{\text{cr},0}\left[\mathrm{in W}\right]=\kappa k_{0}\lambda^{2}/\left(n_{0}\left|\frac{dn}{dT}\right|\right)$ and $L_{d}=k_{0}w_{f}^{2}/2$ ($k_{0}$ is the free-space wavenumber). The fitting is plotted in Figure 8(b) for m=1.75 (d=15 mm) and for A=1/100, which shows a good agreement between simulations and experiments. We consider parameter A, as a correction factor under the applied approximations (aberration-free approximation, initial external focusing condition, $L_{d}$ taken at the linear waist). The $P_{\mathrm{cr},0}$ is a reduced critical power since, in a thermal self-action process, the usual relation of critical power $P_{\mathrm{cr}}\propto \frac{\lambda^{2}}{n_{0}\left|n_{2}\right|}$ (with $n_{2}\propto \frac{dn}{dT}aL_{d}/\left(\kappa k_{0}\right)$ [30]) depends on geometrical characteristics of the beam. For strong absorption ($aL_{d}\gg 1$), $P_{0}$ is independent of a, however m clearly depends on the initial focusing condition, so that $P_{0}\approx P_{\mathrm{cr},0}\sqrt{A\left(m^{2}-1\right)}$. As discussed in [29], this can be understood by the fact that a limited “thin thermal lens” developed at the entrance of a strongly absorbing medium determines balancing of the initial wavefront phase.

Further, we evaluated numerically the divergence of the beam at $P_{0}$ versus various values of a. The divergence was then calculated according to $\left[w_{\sigma^{2}}\left(L\right)-w_{\sigma^{2}}\left(z_{NL}\right)\right]/\left(L-z_{NL}\right).$ A comparison with the experimental results is shown in Figure 8(c). Overall, there is good agreement between the model described by Eqs. (A3) and (A4) and our experimental observations. Accordingly, we conclude that the phenomenological self-trapping by both high repetition rate fs pulses and cw illumination is mainly attributed to steady-state nonlinear thermal self-defocusing of the externally focused beam. The latter effect is caused by optical absorption by a given colloidal solution and governed by the thermal properties of the solvent (here water). Consequently, if the wavelength of the propagating light exactly matches the plasmon resonance of a given nano-colloid, the thermal lensing effect is expected to be further enhanced due to higher absorption cross section of the suspended particles.

**Figure 9:**
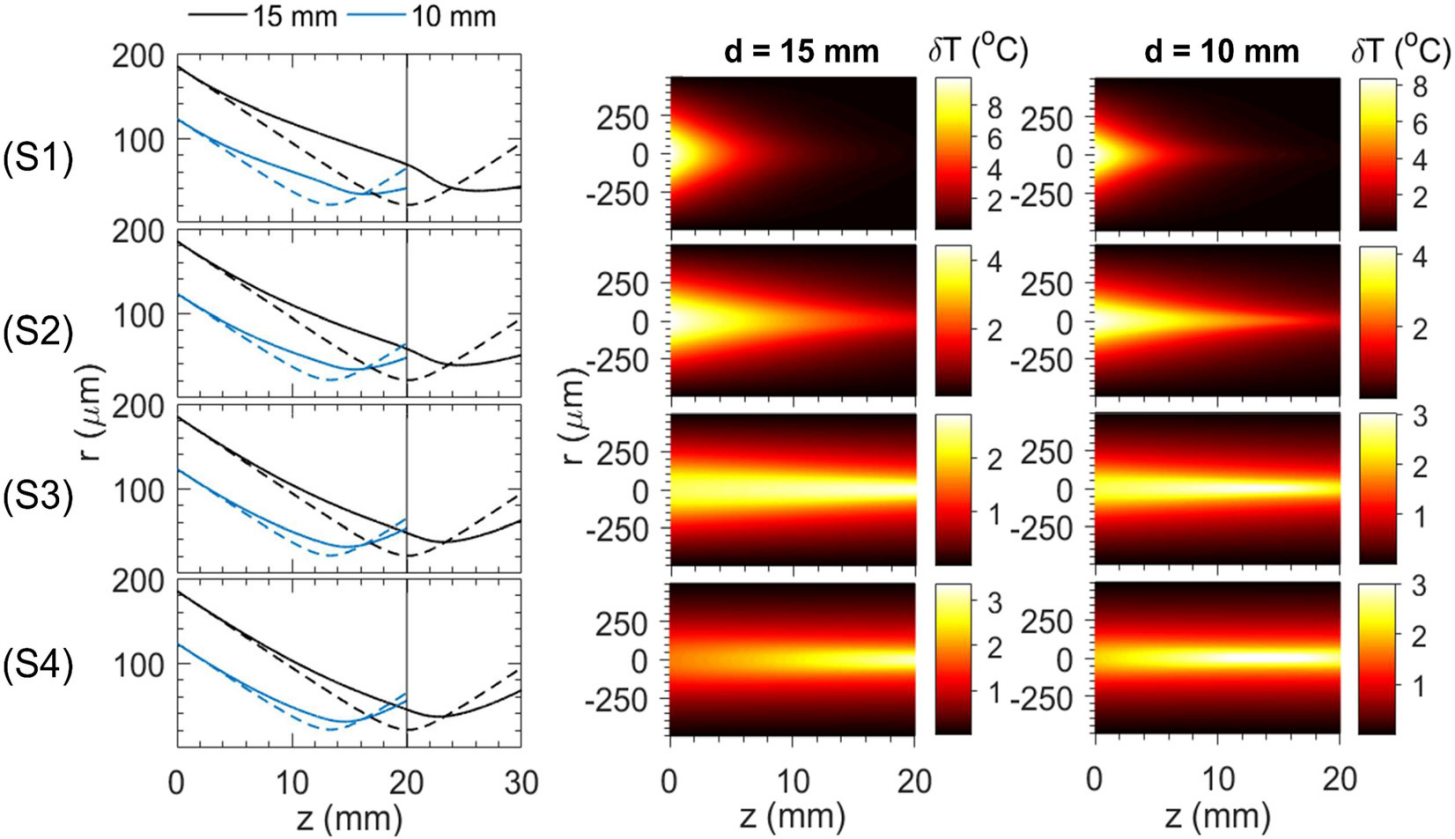
Numerical simulation results at $P=P_{0}$ for (S1) a=2.10, (S2) a=0.84, (S3) a=0.24 and (S4) $a=0.06\;cm^{-1}.$ First column shows the $\sigma^{2}$ beam width as a function of *z* for d=15 mm (black lines) and 10 mm (blue lines). The dashed lines correspond to the linear case (low input power ∼0.1 mW). The second and third column show the calculated spatial temperature profiles at $P=P_{0}$ for d=15 mm and 10 mm, respectively.

In Figure 9, the characteristic-needle like propagation is simulated as a function of propagation *z*. A nonlinear focus is formed due to temperature-induced refractive index changes in the medium that creates the self-channeling effect, reducing θ at the output. In addition, the δT profiles of all four cases of a vary significantly. Transverse temperature gradients are extended at larger radius near the input when a is larger. For small values of a, increased δT is confined in the vicinity of the focus, where ${\left|ℰ\right|}^{2}$ gets higher. This effect has possibly an impact on the development of convective fluid flow due to temperature gradients.

Notably, we have performed an order-of-magnitude comparison between the characteristic times of mass diffusion $t_{d}^{m}$ and heat diffusion $t_{d}^{\mathrm{th}}$ (Supplementary material, Section A.3). We have considered reportedly applied focusing conditions of self-channeling experiments in plasmonic nanocolloids. The calculations showed that, in this context, mass diffusion due to optical forces is a much slower process compared to heat diffusion, so that the latter typically becomes dominant. Nonetheless, the use of high repetition fs pulses exhibits a potential advantage toward mitigation of thermal effects; for instance, if one focuses a beam at a waist of ∼1 μm (to overcome Brownian motion) and applies a repetition rate of ∼1 MHz, $t_{d}^{\mathrm{th}}$ becomes comparable to the time between each pulse $\delta t_{p}$. In this case, heat accumulation can be alleviated. Contrarily, $t_{d}^{m}$ remains much larger than $\delta t_{p}$ so that cumulative optical forces are still expected to trap or repulse particles. In other words, it is possible to engineer an interaction where $t_{d}^{\mathrm{th}}∼\delta t_{p}\ll t_{d}^{m}$ to facilitate gradient force-induced self-trapping in plasmonic nanocolloids by use of fs pulses. However, that would still require considerably high concentrations of particulate material in case of tightly applied focusing [3].

### 3.2 Comparison of resonant nonlinear thermal lensing between fs and cw operation

Experiments on resonant sample S1 demonstrated small differences in the far-field FWHM beam width and divergence between cw and fs operations. The differences may be attributed to the 15% higher absorption coefficient in the case of cw excitation since both the far-field FWHM beam size width and divergence acquire slightly smaller values at the same input power. Nonetheless, at input power of ∼80 mW (well above $P_{0}$), an increase of the divergence of the central Airy disk was observed in cw operation. This fact, in conjunction with the distinct features in the dynamics of the beam profile for the two laser operation modes (Figure 7) indicate an additional contribution to thermal aberration of the beam, presumably due to convective heat transfer.

The breakup of the first diffraction ring, observed in fs and cw operation at P ∼ 140 and ∼120 mW respectively, exhibits small yet important differences in its dynamics and features. The effect itself bears similarities with the break-up of optical vortices propagating in colloidal media [31]. In the latter case, break-up has been attributed to azimuthal modulation instability due to the exponential grown of a perturbation with an orbital angular momentum of specific charge [31, 32]. Here, we used an elliptical, astigmatic Gaussian beam, which is known to possess orbital angular momentum [33]. Additionally, it is possible that transverse convective currents (see for example in Figure 3, horizontal setup, of Reference [34]) contribute to wavefront twisting (in addition to downwards translation). Accordingly, we consider that here, an azimuthal modulation instability led to the first diffraction ring breakup much like for the case of an optical vortex.

**Figure 10:**
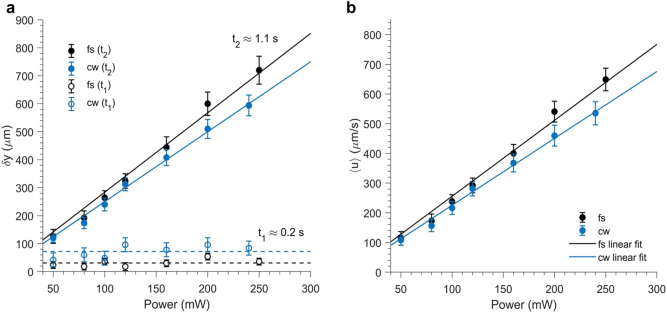
Comparison of (a) the displacement δy, and (b) the average velocity 〈u〉 of the beam profile due to convection under fs and cw operation on sample S1 and d=15 mm as a function of input power. The δy values are shown for two different times $t_{1}$ and $t_{2}$ after the opening of the shutter. The solid lines are linear fittings and the dashed lines in (a) show the average value of data taken for $t_{1}$ at each case. The 〈u〉 values are calculated for time $t_{2}$ after the opening of the shutter.

We analyzed time-resolved images of the beam profile for both fs and cw operation as a function of the input power. Figure 10(a) shows the recorded vertical displacement δy of the core of the beam after the opening of the shutter. We recorded the value of δy as a function of the input power for both cases after $t_{1}\approx 0.2\;s$, when the beam is marginally displaced, and after a time delay $t_{2}\approx 1.1$ s, when the beam appears to decelerate at its final position. The results clearly indicate consistently smaller δy at early times ($t_{1}\approx 0.2\;s)$ in the case of fs operation as compared to cw operation. In addition, δy after $t_{2}$ is higher for fs operation, suggesting that the beam was displaced with a higher average velocity 〈u〉 under the induced convective flow within $t_{2}$.

Balancing the forces of buoyancy and viscous drag force, leads to an estimation of the average downward velocity of the induced flow [28]

(3) $$〈u〉=\frac{a_{V}g\pi w_{0}^{2}\delta Τ}{16\mu }$$

where $a_{v}\approx 2.1\times 10^{-4}\;{}^{\circ}C^{-1}$ is the thermal expansion coefficient of the solvent (water), g is the gravity acceleration, $w_{0}$ is the input beam width and $\mu \approx 0.8\times 10^{-6}\;m^{2}/s$ denotes the kinematic viscosity of the solvent (water). Assuming that at the entrance of the medium δΤ∼8 °C, as seen in the simulations for $P_{0}∼40\;\mathrm{mW}$ (Figure 9), we find 〈u〉≈130 μm/s, in fair agreement with our observations (Figure 10(b)). Noting that $\delta Τ\approx \frac{\alpha Ιw_{0}^{2}}{\kappa }=2\frac{aP}{\pi \kappa }$ the flow velocity can be written in the form

(4) $$〈u〉=\frac{aa_{V}gw_{0}^{2}}{8\mu \kappa }P$$

Eq. (4) shows that the relation between 〈u〉 and P is linear. In addition, the slope of this relation depends linearly on a. Noting that every parameter besides a remains the same for the two laser operation modes, one expects higher 〈u〉 under cw operation, which is not the case. The effect implicates a difference in the combined thermal conduction and convection heat transfer between the two operation modes, which can be expressed by a δT-dependent, overall heat transfer coefficient U(δΤ). Accounting that heat conduction and convection act in series along *y* axis, the overall heat transfer coefficient U reads

(5) $$\frac{1}{U\left(\delta Τ\right)S}=\frac{1}{h\left(\delta Τ\right)S}+\frac{w_{0}}{\kappa S}$$

where h(δΤ) denotes the convection heat transfer coefficient. We also scale the characteristic area $S∼\frac{w_{0}}{a}$, so that $\frac{1}{Uw_{0}}=\frac{1}{hw_{0}}+\frac{1}{\kappa }$. Eq. (4) is rewritten as

(6) $$〈u〉\approx \frac{aa_{V}gw_{0}}{8\mu U}P$$

Eq. (6) shows that for ${\left(\frac{U}{a}\right)}_{\mathrm{cw}}>{\left(\frac{U}{a}\right)}_{\mathrm{fs}}$ the value of 〈u〉 is reduced for the cw case as seen experimentally. Under an oversimplified approach of constant U for both modes, we estimate $1.14{〈u〉}_{\mathrm{cw}}∼{〈u〉}_{\mathrm{fs}}$ (linear fit data of Figure 10(b)). Accounting also for $a_{\mathrm{cw}}∼1.15a_{\mathrm{fs}}$, we find through Eq. (6) that ${\left(U\right)}_{\mathrm{cw}}∼1.31{\left(U\right)}_{\mathrm{fs}}$, showing that the overall heat transfer coefficient in the fs case is significantly lower. Notably, for P≥80 mW, the h convection coefficient in principle increases due to its dependence on local δΤ [35], however, at a different rate between the two modes as shown in Figure 10. For P<80 mW, it is almost zero, so that ${〈u〉}_{\mathrm{cw}}\approx {〈u〉}_{\mathrm{fs}}$.

Overall, strong thermal aberrations at increased powers, appear to be limited in the case of fs illumination, which is evident on (i) the analysis just described, (ii) the thermal blooming features shown in Figure 6, and (iii) the weaker stochastic motion of the beam (Supplementary video). A possible explanation can be given by the fact that, under excitation by fs pulses, temperature rise is highly confined in the vicinity of the nanoparticles [27]. The temperature increase profile decreases rapidly in space away from the surface of the particle as $\propto r^{-3}$ (ideal case of point source), as opposed to the $\propto r^{-1}$ dependency for cw operation [27]. This is because in the fs case the deposited energy, absorbed by the plasmon mode after each pulse, decays exponentially in time during thermalization of the electrons with the phonon subsystem of the particle before it is transferred through the particle interface to the surrounding solvent. Accordingly, under cw excitation and at short time delays, temperature increase in the medium by heat conduction is less localized compared to the case of fs operation. Homogeneous temperature increase is established faster in the medium so that the effect induces convective currents and beam deflection at slightly shorter time delays ($t_{1}$) compared to fs operation at a wide range of powers (Figure 10(a)), which confirms that the effect cannot be attributed to linear absorption difference. Effectively, in the fs case, overall thermal resistance due to convective heat transfer is higher at increasing P, which affects the vertical deflection of the beam, break-up dynamics and thermal distortion (blooming).

## 4 Conclusions

We have studied phenomenological self-trapping of high repetition rate fs laser pulses in plasmonic nanocolloids of varying plasmon resonance under typically reported external focusing conditions. The excitation regime resulted in cumulative effects, exhibiting a quasi-cw behaviour. Experimental observations of the far-field beam width and divergence indicated similarity for all samples up to a critical power. They further implied phenomenological self-trapping due to stationary, photo-absorption thermal defocusing of an externally focused beam, for both cw and fs excitation. A good agreement between numerical experiments and the experimental observations supported the foresaid model suggesting that the effect can be generally observed in any absorbing medium.

An important element of the studied effect in a soft-matter system is the induction of convective currents that causes beam downward deflection in a horizontal illumination configuration. Under resonant fs and cw excitation of plasmonic colloids we observed that beam deflection was further accompanied by beam spatial mode break-up at increasing input powers, most likely due to the ellipticity of the beam. By analyzing the dynamics of the effect for both cases, we conclude that under fs excitation, convective heat transfer appears to be, relatively to the cw excitation, reduced in magnitude and decoupled in time from conductive heat transfer. This is presumably because fs illumination, as opposed to cw, results typically in spatial temperature increase confinement near the particles. Effectively, delayed beam break-up and reduced beam axial asymmetry due to thermal blooming at increased power are observed.

Finally, according to our analysis, we conclude that the (high) repetition rate of fs pulses in conjunction with tight focusing (high numerical aperture) constitute dominant parameters for alleviating thermal effects and promoting observation of nonlinear self-trapping induced by gradient optical forces in plasmonic nanocolloids.

## Supplementary Material

Supplementary Material

## References

[1] Brzobohatý O., Chvátal L. s., Jonáš A. (2019). Tunable soft-matter optofluidic waveguides assembled by light. *ACS Photonics*.

[2] Fardad S., Mills M. S., Zhang P., Man W., Chen Z., Christodoulides D. (2013). Interactions between self-channeled optical beams in soft-matter systems with artificial nonlinearities. *Opt. Lett.*.

[3] Lamhot Y., Barak A., Peleg O., Segev M. (2010). Self-trapping of optical beams through thermophoresis. *Phys. Rev. Lett.*.

[4] Man W., Fardad S., Zhang Z. (2013). Optical nonlinearities and enhanced light transmission in soft-matter systems with tunable polarizabilities. *Phys. Rev. Lett.*.

[5] Sun J., Silahli S. Z., Walasik W., Li Q., Johnson E., Litchinitser N. M. (2018). Nanoscale orbital angular momentum beam instabilities in engineered nonlinear colloidal media. *Opt. Express*.

[6] Ortega B.A., Brambila E. C., López Gayou V. (2019). Light control through a nonlinear lensing effect in a colloid of biosynthesized gold nanoparticles. *J. Mod. Opt.*.

[7] Fardad S., Salandrino A., Heinrich M., Zhang P., Chen Z., Christodoulides D. N. (2014). Plasmonic resonant solitons in metallic nanosuspensions. *Nano Lett.*.

[8] Kelly T. S., Ren Y.-X., Samadi A., Bezryadina A., Christodoulides D., Chen Z. (2016). Guiding and nonlinear coupling of light in plasmonic nanosuspensions. *Opt. Lett.*.

[9] Ortega A. B., Torres-González F. E., Gayou V. L. (2021). Guiding light with singular beams in nanoplasmonic colloids. *Appl. Phys. Lett.*.

[10] Ren Y.-X., Kelly T. S., Zhang C., Xu H., Chen Z. (2017). Soliton-mediated orientational ordering of gold nanorods and birefringence in plasmonic suspensions. *Opt. Lett.*.

[11] Reyna A. S., Boudebs G., Malomed B. A., de Araújo C. B. (2016). Robust self-trapping of vortex beams in a saturable optical medium. *Phys. Rev.*.

[12] Reyna A. S., de Araújo C. B. (2016). Guiding and confinement of light induced by optical vortex solitons in a cubic–quintic medium. *Opt. Lett.*.

[13] Shvedov V., Cyprych K., Salazar-Romero M. Y., Izdebskaya Y., Krolikowski W. (2018). Nonlinear propagation and quasi self-confinement of light in plasmonic resonant media. *Opt. Express*.

[14] Xiang Y., Liang G., Alvaro P. (2021). Resonant optical nonlinearity and fluorescence enhancement in electrically tuned plasmonic nanosuspensions. *Adv. Photon. Res.*.

[15] Xu H., Alvaro P., Xiang Y. (2019). Plasmonic resonant nonlinearity and synthetic optical properties in gold nanorod suspensions. *Photon. Res.*.

[16] Bezryadina A., Hansson T., Gautam R. (2017). Nonlinear self-action of light through biological suspensions. *Phys. Rev. Lett.*.

[17] Gautam R., Bezryadina A., Xiang Y. (2020). Nonlinear optical response and self-trapping of light in biological suspensions. *Adv. Phys. X*.

[18] Gautam R., Xiang Y., Lamstein J. (2019). Optical force-induced nonlinearity and self-guiding of light in human red blood cell suspensions. *Light Sci. Appl.*.

[19] Perez N., Chambers J., Chen Z., Bezryadina A. (2021). Nonlinear self-trapping and guiding of light at different wavelengths with sheep blood. *Opt. Lett.*.

[20] Xin H., Li Y., Liu X., Li B. (2013). Escherichia coli-based biophotonic waveguides. *Nano Lett.*.

[21] Litchinitser N. M. (2018). Nonlinear optics in metamaterials. *Adv. Phys. X*.

[22] Agate B., Brown C., Sibbett W., Dholakia K. (2004). Femtosecond optical tweezers for in-situ control of two-photon fluorescence. *Opt. Express*.

[23] Falconieri M. (1999). Thermo-optical effects in z-scan measurements using high-repetition-rate lasers. *J. Opt. Pure Appl. Opt.*.

[24] Jiang Y., Narushima T., Okamoto H. (2010). Nonlinear optical effects in trapping nanoparticles with femtosecond pulses. *Nat. Phys.*.

[25] Liu T.-H., Chiang W.-Y., Usman A., Masuhara H. (2016). Optical trapping dynamics of a single polystyrene sphere: continuous wave versus femtosecond lasers. *J. Phys. Chem. C*.

[26] Mian S. M., McGee S. B., Melikechi N. (2002). Experimental and theoretical investigation of thermal lensing effects in mode-locked femtosecond z-scan experiments. *Opt. Commun.*.

[27] Baffou G., Rigneault H. (2011). Femtosecond-pulsed optical heating of gold nanoparticles. *Phys. Rev. B*.

[28] Smith D. C. (1977). High-power laser propagation: thermal blooming. *Proc. IEEE*.

[29] Khachatrian A., Sukhorukov A. (1971). Some aspects of thermal self-focusing. *Opto-Electronics*.

[30] Boyd R. W. (2020). *Nonlinear Optics*.

[31] Silahli S. Z., Walasik W., Litchinitser N. M. (2015). Necklace beam generation in nonlinear colloidal engineered media. *Opt. Lett.*.

[32] Vincotte A., Bergé L. (2005). Femtosecond optical vortices in air. *Phys. Rev. Lett.*.

[33] Courtial J., Dholakia K., Allen L., Padgett M. (1997). Gaussian beams with very high orbital angular momentum. *Opt Commun.*.

[34] Rusconi R., Isa L., Piazza R. (2004). Thermal-lensing measurement of particle thermophoresis in aqueous dispersions. *J. Opt. Soc. Am. B*.

[35] Singhal S., Goswami D. (2019). Thermal lens study of nir femtosecond laser-induced convection in alcohols. *ACS Omega*.

