# Supplementary material for: Nonlinear thermal lensing of high repetition rate ultrafast laser light in plasmonic nano-colloids
Source: Nanophotonics. 2022 Jan 18;11(5):1051–62. doi: 10.1515/nanoph-2021-0775 (PMC8997721; doi:10.1515/nanoph-2021-0775)
Supplement: Supplementary file 1 — Supplementary Material [file j_nanoph-2021-0775_suppl.docx]

Nonlinear thermal lensing of high repetition rate ultrafast laser light in plasmonic nano-colloids – Supplemental Material

**Contents**

[A1. Methods 2](#_Toc90034398)

[A1.1 Far-field beam width and divergence 2](#_Toc90034399)

[A1.2 Sample preparation and optical characterization 3](#_Toc90034400)

[A1.3 Numerical simulations 4](#_Toc90034401)

[A2 Simulation results for the case d = 10 mm and comparison with experiments. 6](#_Toc90034402)

[A3 Characteristic times of mass diffusion and nonlinear thermal lensing 6](#_Toc90034403)

[References 7](#_Toc90034404)

# A1. Methods

## A1.1 Far-field beam width and divergence

In this section, we describe the experimental setup for the evaluation of the far-field FWHM beam size and divergence due to phenomenological self-trapping in plasmonic nano-colloids, shown in Figure A1(a).

We used a Ti:Sapphire femtosecond oscillator, running at 76 MHz repetition rate. The system initially delivers >35 fs pulses at wavelength $\lambda=800 nm$ (measured FWHM bandwidth of ~35 nm). The maximum average output power is ~300 mW. The system can operate as a cw laser by adjustment of a pair of prisms that control the net group delay dispersion of the cavity, so that the phase will not be locked at an emission bandwidth around 800 nm. The transition of fs to cw operation was monitored on a spectrometer and a fast photodiode. At cw operation the system delivers approximately the same average power.


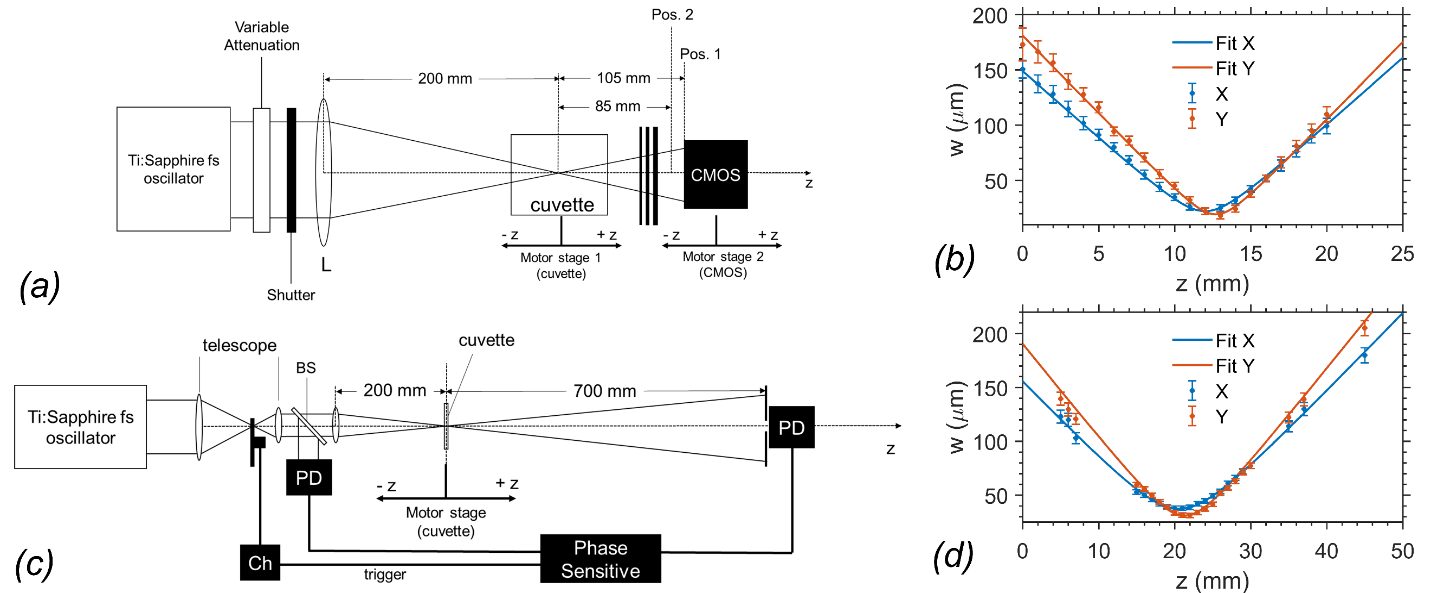


*Figure A1. (a) The experimental setup for the evaluation of far-field beam size and divergence variation as a function of input power in plasmonic nanocolloids. (b) Beam width characterization in air near the geometrical focus of lens L for the setup shown in (a). (c) z-scan characterization setup for the examined plasmonic nanocolloids. (d) Beam width characterization in air near the geometrical focus of the z-scan setup shown in (c).*

The input laser beam had an elliptical profile of Gaussian shape along the vertical Y and horizontal X axis that define a plane normal to the propagation direction Z. The initial 1/e^2^ beam radius along the Y axis was $w_{0,Y}$~2.8 mm and along the X axis was $w_{0,X}$~2.4 mm, which yields a beam ellipticity $e\equiv\frac{w_{0,Y}}{w_{0,X}}\approx1.2$. The beam passes through a variable attenuator and a shutter (the latter is used only for time-resolved experiments) and is focused by a positive lens L of focal distance 200 mm. The Rayleigh range $z_{R,Y}$, $z_{R,X}$ of the focused beam in air was determined by a knife edge method, along Y and X axes [Fig. A1(b)]. We evaluated $z_{R,Y}\sim1.4$ mm, $z_{R,X}\sim1.8$ mm. The propagation factor $M_{Y}^{2}\approx M_{X}^{2}\sim1.08$ was evaluated by fitting equations

|  | $w_{Y}\left( z \right)=w_{f,Y}^{ideal}\sqrt{M_{Y}^{2}+{M_{Y}^{2}\left( \frac{z-z_{0,Y}}{{n_{0}z}_{R,Y}} \right)}^{2}}$ | (A1a) |
| --- | --- | --- |
|  | $w_{X}\left( z \right)=w_{f,X}^{ideal}\sqrt{M_{X}^{2}+{M_{X}^{2}\left( \frac{z-z_{0,X}}{{n_{0}z}_{R,X}} \right)}^{2}}$ | (A1b) |

on the experimental data, where $w_{f,Y}^{ideal}=\sqrt{\lambda z_{R,Y}/\pi}$, $w_{f,X}^{ideal}=\sqrt{\lambda z_{R,X}/\pi}$ denote the ideal beam waist size, $z_{0,Y}$, $z_{0,X}$ denote the Z coordinates that the beam waist is formed, and $n_{0}$ is the refractive index of the medium. The fitting also provides the 1/e^2^ beam waist radii, $w_{f,Y}\sim19 \mu m$ and $w_{f,X}\sim22 \mu m$. Further, we evaluated the distance $D\equiv z_{0,X}-z_{0,Y}\sim0.7 mm$. The geometrical focus is approximately at the same location as the beam waists (uncertainty of $\sim1mm$).

A 20 mm long cuvette was positioned on a motorized translation stage near the geometrical focus. The distance between the geometrical focus in air and the entrance window of the cuvette $d$ varied (between 5, 10 and 15 mm). The cuvette contained each of the four examined samples (described below). The emerged beam was imaged, as a function of input power, on a CMOS camera, positioned at a motorized stage and translated at two different positions, $\sim85$ mm and $\sim105$ mm apart from the geometrical focus. Neutral density filters, placed before the camera, were used to adjust the power density below saturation. The FWHM size of the beam profile $w_{FWHM}$ in the far-field was evaluated by processing the collected images. The divergence of the beam in the far-field was calculated as $\theta(rad)=\left[ w_{FWHM}\left( 105mm \right)-w_{FWHM}\left( 85mm \right) \right]/\left[ \sqrt{2ln2}\times20mm \right]$, accounting for $w_{FWHM}=w_{\left( 1/e^{2} \right)}\sqrt{2ln2}$. In dynamic experiments, videos were recorded by the camera at a framerate of ~45.06 $s^{-1}$ upon opening of a mechanical shutter (~1 ms) placed before lens L.

## A1.2 Sample preparation and optical characterization

We prepared four samples of different plasmonic nanocolloids: (S1) Au nanorods (purchased by Nanopartz) of 10 nm diameter and 38 nm length with a longitudinal peak around 770 nm. (S2) Au nanorods (purchased by Nanopartz) of 10 nm diameter and 50 nm length with a longitudinal peak around 900 nm. (S3) Au nanospheres (purchased by Nanopartz) of 50 nm diameter with a plasmon peak around 525 nm. (S4) AuAg alloy (15:85) (fabricated in our laboratory [1]) of 40 nm diameter with a plasmon peak around 450 nm. The concentration of samples S1, S2 and S3 were (according to specifications) $CS1=6.73\times{10}^{11} {ml}^{-1}$, $CS2=4.93\times{10}^{11} {ml}^{-1}$ and $CS3=3.97\times{10}^{11} {ml}^{-1}$ respectively. The concentration of sample S4 was evaluated $CS4 \approx3\times{10}^{11} {ml}^{-1}.$

We performed optical transmittance $T$ measurements for all samples, tested on the setup of Figure A1(a), under fs operation. We have measured the input and output power after the $l=20 mm$ long cuvette by subtracting the influence of the cuvette. The samples were positioned in the three foresaid examined $d$ positions so that the transmittance was evaluated each time as a function of power for each sample and $d$. No significant difference was observed at every $d$ for a specific sample, while the transmittance exhibited linear behaviour in the examined power range since the beam was always focused deep into the cuvette (>4 mm). The absorption coefficient $a$ of all samples was evaluated according to $T=-\ln\left( al \right)$.

Further, we implemented a standard z-scan setup to evaluate the thermo-optical coefficient $\frac{dn}{dT}$ of the examined samples, under fs operation [Fig. A1(c)]. A $0.64\times$ telescope was used to reduce the beam size to $w_{0,Y}\sim1.8 mm and w_{0,X}\sim1.5 mm$. A mechanical chopper set at a frequency of 20 Hz and a duty cycle $d_{c}\sim16\%$ was placed on the focus of the telescope, resulting in laser excitation of $8 ms$ every $50 ms$ (sufficient time for heat conduction, see for example references [2, 3]). Right after the telescope, a 50:50 beam-splitter directed half of the beam’s energy onto a reference photodetector and half on a 200 mm focal length lens. We performed a knife edge characterization of the focused beam in air [the results are shown in Figure A1(d)]. We evaluated (according to definitions shown above), $z_{R,Y}\sim3.5$ mm, $z_{R,X}\sim5.1$ mm, $w_{f,Y}\sim30 \mu m$ and $w_{f,X}\sim36 \mu m$ and $D\sim0.7 mm$. The samples were contained in an optical cuvette of length $l=2 mm$ so that $l<z_{R,Y}, z_{R,X}$. The latter was positioned in a motorized stage and scanned over a length of ~70 mm. An apertured photodetector was placed ~700 mm apart from the focal plane, yielding transmittance $S\sim0.01$ of the central portion of the impinged beam profile. To better read the weak signal, the photodetector was connected to a lock-in amplifier, which was triggered by the chopper’s frequency. Accordingly, to determine $\frac{dn}{dT}$ we have used the relations (for 1-photon absorption) [4, 5]:

|  | $\Delta T_{p-v}=0.405\left\vert\Delta\Phi\right\vert$ | (A2a) |
| --- | --- | --- |
|  | $\frac{dn}{dT}=\frac{\lambda\kappa}{\alpha Pd_{c}l_{eff}}\Delta\Phi$ | (A2b) |


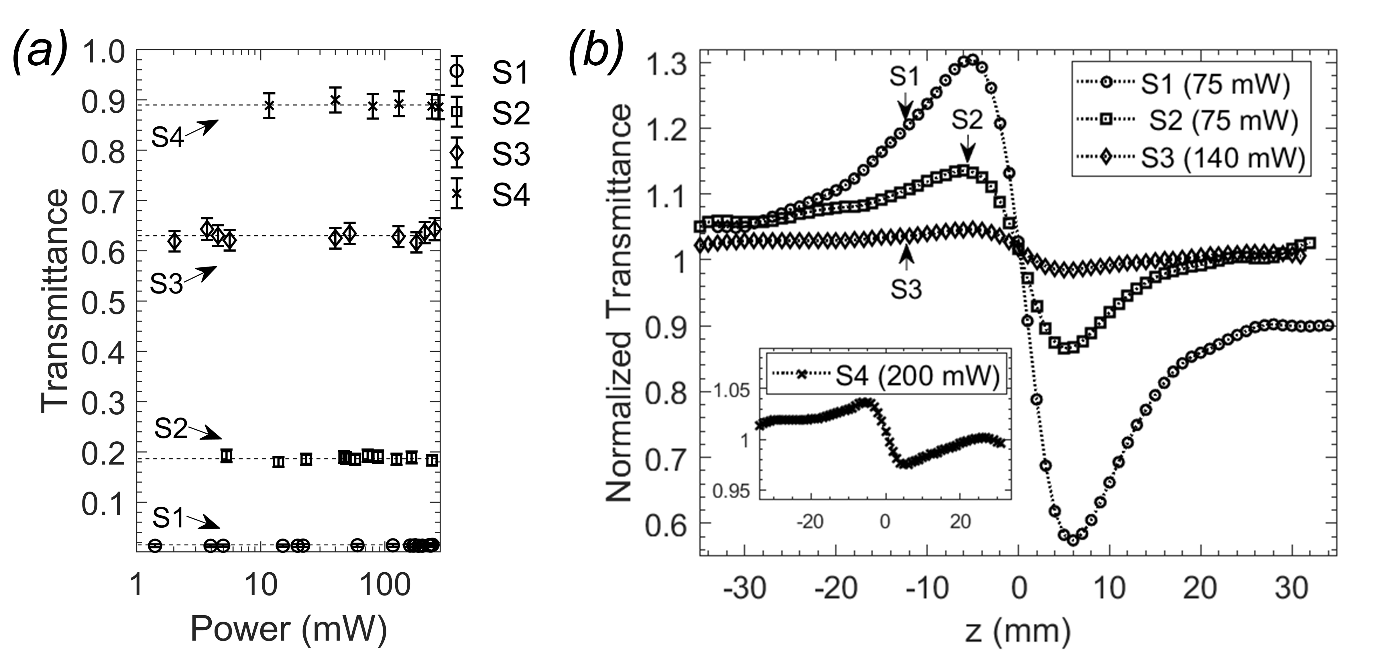


*Figure A2 (a) Optical transmittance measurements on the examined plasmonic nanocolloids as a function of $P$ and $d=15 mm$. The measurements were performed on the setup shown in Figure 1(a). (b) z-scan signals on the examined plasmonic nanocolloids. Both optical transmittance and z-scan measurements shown here were performed by fs pulsed irradiation.*

where $\Delta T_{p-v}$ is the normalized recorded peak to valley transmittance difference, $\Delta\Phi$ is the deduced nonlinear phase shift from Eq. A2a, $\kappa$ is the thermal conductivity of water, $a$ is the absorption coefficient of the samples, $P$ is the average optical power of the fs laser after the mechanical chopper and the effective length reads $l_{eff}=\left( 1-e^{-al} \right)/a$. Our measurements confirm a negative thermal nonlinearity for all samples (even far from the resonance) and the value of $\frac{dn}{dT}$ does not vary significantly since it is an intrinsic property. The volumetric filling factor $f_{v}$ of all samples is $\ll1$ so that $\frac{dn}{dT}$ is governed by the solvent (water). All values are reported in Table 1.

## A1.3 Numerical simulations

We compare our experimental observations with a thermal lensing model. We performed numerical simulations of the steady-state (with respect to heat conduction) nonlinear Schrodinger equation with a thermal nonlinearity and linear absorption. The model reads:

|  | $2ik_{0}\partial_{z}\mathcal{E+}\Delta_{\perp}\mathcal{E}+\frac{2k_{0}^{2}}{n_{0}}\frac{dn}{dT}\delta T\mathcal{E-}ik_{0}a\mathcal{E}=0$ | (A3a) |
| --- | --- | --- |
|  | $-\kappa\Delta_{\perp}\left( \delta T \right)=\alpha\left\vert\mathcal{E} \right\vert^{2}$ | (A3b) |

Where $z$ is the propagation coordinate, $k_{0}={2\pi n_{0}}/\lambda$ is the wavenumber of the central laser wavelength $\lambda$ and $\Delta_{\perp}$ is the transverse Laplacian in cylindrical coordinates (for radial coordinate $r$). The initial beam profile was defined to match the experimental setup of the focused beam at the entrance of the cuvette as

|  | $\mathcal{E}\left( r,0 \right)=\sqrt{\frac{2P_{in}}{\pi w_{0}^{2}}}exp\left( -\frac{r^{2}}{w_{0}^{2}}-i\frac{k_{0}r^{2}}{2R} \right)$ | (A4) |
| --- | --- | --- |

where $P_{in}$ is the input power, $w_{0}$ is the initial 1/e^2^ beam radius, $R=n_{0}d-z_{R}^{2}/\left( n_{0}d \right)$ is the radius of curvature. We choose $z_{R}=n_{0}z_{R,X}$ to be the Rayleigh length, (experimental $z_{R,X}$) and we performed simulations according to the measured values of the beam width along the X axis for simplicity (all simulations can be extended by accounting experimental measurements over Y axis). We examined two cases of $d$ (10 and 15 mm), where phenomenological self-trapping is more pronounced as opposed to $d=5mm$, for the values of $a$ of each sample shown in Table 1. The waist is formed at a position $n_{0}\times d$ from the entrance of the cuvette due to the difference in the index of refraction. Since this value is approximately equal to the length of the cuvette for the case $d=15$ mm, we have solved equations (A3) assuming a 3 cm medium length instead of 2 cm (the latter is the actual length of the cuvette) for convenience in comparing the nonlinear focus position and beam profile at the output with the ones of $d=10$ mm case. The physical picture is not affected since temperature gradients developed for z<2cm determine self-action for both cases (see for example Fig. 8). After integration of equations (A3), we evaluated the $\sigma^{2}$ beam radius, which is equal to the 1/e^2^ definition for a Gaussian spatial profile, yet more meaningful when the beam profile undergoes thermal lens distortion. A comparison of the experimental fit along X axis and the numerical simulations at very low $P_{in}<0.1 mW$(linear regime) in a medium of refractive index $n_{0}=1.33$ is shown in Figure A3 for two values of $d$ (10 and 15 mm). The parameters that were used for the solution of (A3)-(A4) are shown in Table A1.


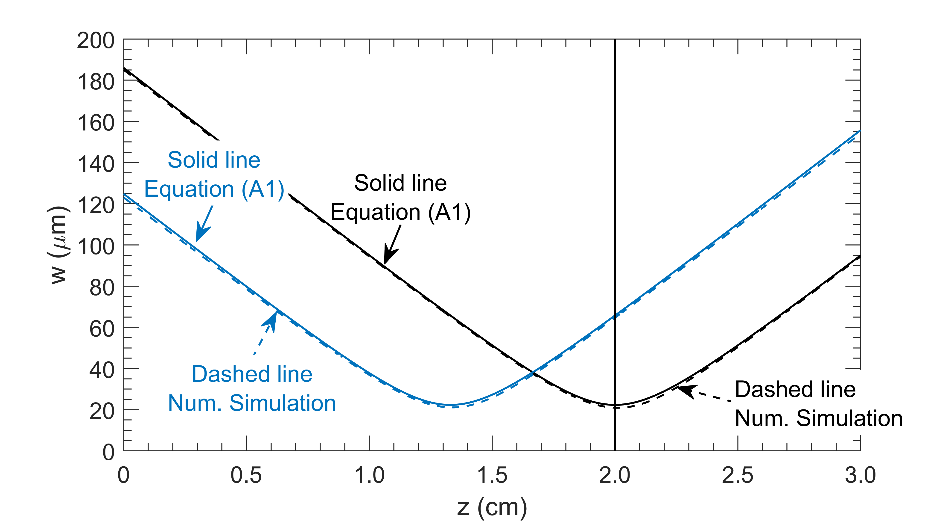


*Figure A3. Comparison of analytical equation (A1b) and the $\sigma^{2}$ beam width determined by solution of equations (A3)-(A4). Equation (A1b) of the $w_{X}(z)$ is plotted for $z_{0,X}=n_{0}\times d=13.3 mm \left( d=10 mm \right)$ (solid blue line) and $z_{0,X}=20.0 mm$ $\left( d=15 mm \right)$ (solid black line) for the experimentally determined $M_{X}^{2}$ $w_{f,X}^{ideal}$, $z_{R,X}$ and for $n_{0}=1.33$. The solution of equations (A3)-(A4) was determined for $n_{0}\times d=13.3 mm$ (dashed blue line) and $n_{0}\times d=20.0 mm$ (dashed black line), for very low powers and for the corresponding parameters shown in Table 2. The solid vertical line shows the output window of the cuvette.*

*Table A1. Parameters used for the solution of equations (A3)-(A4), which describes stationary photo-absorption thermal defocusing of a focused beam for the examined colloids and the experimental conditions applied in this study.*

## A2 Simulation results for the case d = 10 mm and comparison with experiments.


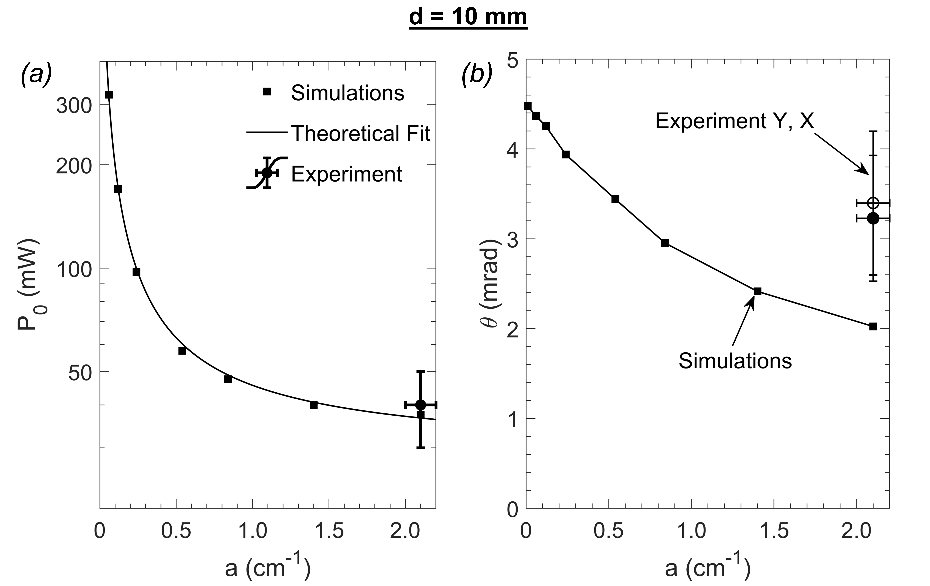


*Figure A4. Results of numerical simulations based on the nonlinear Schrödinger equation with a thermal nonlinearity (equations (A3)-(A4)) and comparison with experimental observations for $d=10 mm$. (a) Comparison of $P_{0}$(a) by numerical simulation (squares), fitting of analytical expression (2) for m=1.5 (solid line) and experiments (circles). (b) Comparison of $\theta\left( \alpha\right)$ between numerical simulations (squares) and experimental measurements (circles).*

# A3 Characteristic times of mass diffusion and nonlinear thermal lensing

In most reports on self-trapping in colloidal solutions (plasmonic or not), the experimental setup is rather similar: a cw laser beam is focused on a beam width (FWHM) of ~5-25 μm, typically several millimeters from the entrance of an optical cuvette (2-10 cm long) that contains the samples. Several studies have attributed the effect to phase modulation due to a gradient-force-induced, local concentration change of suspended particles. The supporting theoretical formalism imposes that a steady state is reached, governed by the mass diffusion equation [6]:

|  | $\frac{\partial\rho}{\partial t}+\nabla\cdot\left( \rho\vec{v}-D\nabla\rho\right)=0$ | (A5) |
| --- | --- | --- |

Where $\rho$ denotes the particle concentration, $D$ the mass diffusion coefficient and $\vec{v}$ the particle convective velocity.

One can perform an order-of-magnitude estimate of the characteristic time of this process, by replacing $\nabla^{2}\to1/R^{2}$ and ${\partial\rho}/{\partial t}\to\rho/t_{d}^{m}$, where $R$ is a characteristic length (usually the beam spot) and $t_{d}^{m}$ is the characteristic time of mass diffusion. Equation (A5) leads accordingly to the approximation (after appropriate treatment of the term $\nabla\cdot\rho\vec{v}$ presented in [6]):

|  | $t_{d}^{m}\geq\frac{R^{2}}{\left\vert D-D\frac{a\mathcal{E}\mathcal{E}^{*}}{4k_{B}T} \right\vert}$ | (A6) |
| --- | --- | --- |

Where $a$ is the particle polarizability $\mathcal{E}$ is the complex amplitude of the applied electric field, $k_{B}$ is Boltzmann’s constant and $T$ denotes the local temperature. From relation (A6) we observe that a short value of $t_{d}^{m}$ is managed by the polarizability term $D\frac{a\mathcal{E}\mathcal{E}^{*}}{{4k}_{B}T}$, since the mass diffusion coefficient of nanoparticles of radius ~25-250 nm is relatively small (10-1 $\mu m^{2}/s$ respectively), if we account for Stokes-Einstein’s relation $D=k_{B}T/\left( 6\pi\eta r \right),$ where $\eta$ is the dynamic viscosity of the solvent and $r$ is the radius of the particle. Thus, for a particle of polarizability $a\sim3V\varepsilon_{0}$, where $V$ denotes the volume of the particle and $\varepsilon_{0}$ is the vacuum permittivity, and $\mathcal{E}\mathcal{E}^{*}\sim2P/\left( \pi R^{2}\varepsilon_{0}c \right)$, where $P$ is the optical power and $c$ the speed of light, we find that

|  | $t_{d}^{m}\gtrsim\frac{R^{4}}{r^{2}}\frac{3\pi\eta c}{P}$ | (A7) |
| --- | --- | --- |

Assuming that $P$ of 1 W is delivered at a focused spot of 1/e^2^ radius $R$ of 5 μm (or ~5.9 μm FWHM width), without losses, inside an aqueous solution ($\eta\approx9\times{10}^{-4}Pa\cdot s$) of 25 nm radius plasmonic particles, we find $t_{d}^{m}\gtrsim2.5 s$, which significantly exceeds theoretical characteristic times of thermal diffusion. Indeed, if we assume an irradiation volume characterized by two axis, a short axis ~R and a long axis equal to the Rayleigh length $n_{0}\pi R^{2}/\lambda$ (assume n_0_ = 1.33 of water, λ = 800 nm) we can define the thermal diffusion length $L_{d}=V/S\sim R$, where $V$ and $S$ are the volume and surface area of the ellipsoid. Then a characteristic thermal diffusion time is defined as [7]

|  | $t_{d}^{th}\sim\frac{L_{d}^{2}}{4k}$ | (A8) |
| --- | --- | --- |

Where $k$ is the thermal diffusivity of the medium (=1.46×10^-7^ m^2^/s for water). Applying $R$ of 5 μm, we find that $t_{d}^{th}\gtrsim7.9 \mu s$. Note that the value of $t_{d}^{m}$ is increasing dramatically as $R$ increases, limited by the characteristic time of Brownian motion. While this problem can potentially be balanced by using larger particles, it comes at the expense of significant increase in absorption and scattering cross-sections for the case of plasmonic particles, which further enhances thermal or radiation pressure effects.

*
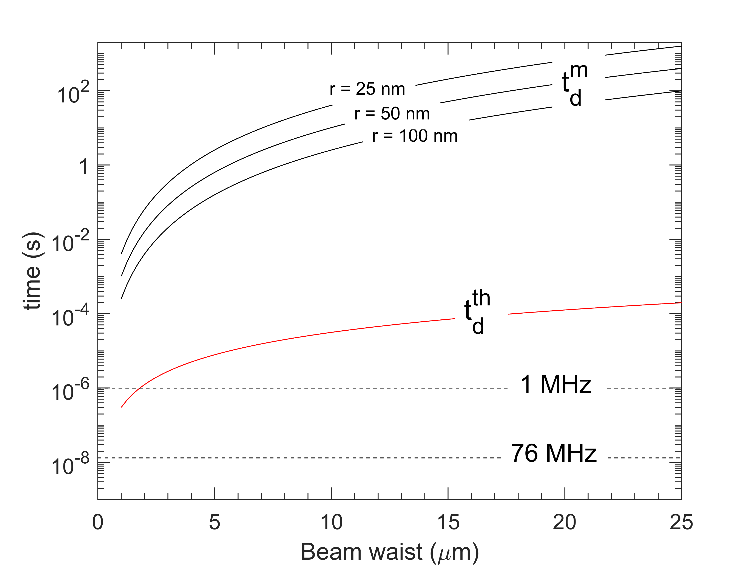
*

Figure A5. Characteristic times for heat diffusion (red curve) and mass diffusion (black curves) described by equations (A7) and (A8) respectively as a function of beam waist radius in the case of aqueous suspension of spherical nanoparticles and an input power of 1 W. The black horizontal dashed lines indicate ${\delta t}_{p}$ for illumination by use of high repetition rate fs pulses at two different repetition rates.

Nonetheless, high repetition fs illumination offers a potential advantage toward the mitigation of thermal effects. If tighter focusing conditions ($R\lesssim2\mu m$, high numerical apertures, limit aberrations) are applied, it is possible that thermal effects are alleviated while maintaining the possibility of mass transport via gradient forces. This is because the time between two pulses ${\delta t}_{p}$ becomes comparable to $t_{d}^{th}$ so that temperature increase in the medium will be negligible on the next pulse. Contrarily, $t_{d}^{m}$ remains much larger than ${\delta t}_{p}$, ensuring a cumulative effect of exerted optical force. For a smaller repetition rate of ~1 MHz, the time between pulses further approaches $t_{d}^{th}$ so that such a scenario becomes plausible. Figure A5 summarizes the conclusions of the foregoing example.

# References

[1] D. Rioux, M. Meunier, Seeded growth synthesis of composition and size-controlled gold–silver alloy nanoparticles, The Journal of Physical Chemistry C, 119 (2015) 13160-13168.

[2] M. Falconieri, G. Salvetti, Simultaneous measurement of pure-optical and thermo-optical nonlinearities induced by high-repetition-rate, femtosecond laser pulses: application to CS2, Applied physics B, 69 (1999) 133-136.

[3] A. Gnoli, L. Razzari, M. Righini, Z-scan measurements using high repetition rate lasers: how to manage thermal effects, Optics express, 13 (2005) 7976-7981.

[4] M. Falconieri, Thermo-optical effects in Z-scan measurements using high-repetition-rate lasers, Journal of Optics A: Pure and Applied Optics, 1 (1999) 662.

[5] M. Sheik-Bahae, A.A. Said, E.W. Van Stryland, High-sensitivity, single-beam n 2 measurements, Optics letters, 14 (1989) 955-957.

[6] R. El-Ganainy, D. Christodoulides, C. Rotschild, M. Segev, Soliton dynamics and self-induced transparency in nonlinear nanosuspensions, Optics express, 15 (2007) 10207-10218.

[7] G. Baffou, Thermoplasmonics: Heating Metal Nanoparticles Using Light, Cambridge University Press, Cambridge, 2017.
